# Challenges to Video Visits for Patients With Non–English Language Preference: A Qualitative Study

**DOI:** 10.1001/jamanetworkopen.2024.57477

**Published:** 2025-02-12

**Authors:** Marianna Kong, Francine Rios-Fetchko, Madelyn Olmos-Rodriguez, Linda Branagan, Bradley Iott, Therese Chan Tack, Carol Yarbrough, Kevin Grumbach, Alicia Fernandez

**Affiliations:** 1 Department of Family and Community Medicine, University of California, San Francisco; 2 University of California, San Francisco; 3 University of California, Irvine; 4 University of Michigan, Ann Arbor; 5 Department of Medicine, University of California, San Francisco

## Abstract

**Importance:**

Patients with non–English language preference (NELP) participate in video visits considerably less often than their English-speaking counterparts. Understanding the capability, opportunities, and motivation of patients with NELP to use video visits is key to determining how this technology may benefit their care.

**Objective:**

To investigate the perceptions of patients with NELP regarding the facilitators and barriers to usage of video visits.

**Design, Setting, and Participants:**

This qualitative analysis of semistructured interviews of patients with NELP from an ambulatory clinic network of a large, urban, academic health system in California serving a linguistically diverse population was conducted from November 2022 to September 2023. Electronic health data were used to identify Spanish- and Cantonese-speaking patients with at least 1 clinic visit but no video visits in the prior 12 months. Data were analyzed from September 2023 to February 2024.

**Main Outcomes and Measures:**

One-on-one semistructured interviews were conducted by telephone to capture perceptions on facilitators and barriers to video visit use. Interviews were audio-recorded until reaching thematic saturation, deidentified, transcribed and translated, and analyzed using rapid qualitative analysis. Common themes were identified and matched to relevant domains of the capability, opportunities, and motivation model.

**Results:**

Of the 31 patients successfully contacted by telephone, 27 participants (mean [SD] age, 66 [15] years; 18 women [67%]), including 16 Spanish-speaking and 11 Cantonese-speaking participants, were interviewed. Four major themes emerged, including (1) video visits creating additional communication challenges and potentially exacerbating communication difficulties for patients who face language barriers; (2) video visits perceived as having some drawbacks for medical evaluations as well as some appealing benefits; (3) limited digital literacy, device and data access, and non–user-friendly video visit processes as important barriers; and (4) in-person teaching, simpler technologic processes, opportunities to repetitively practice video use, troubleshooting support, and language-concordant instructions, clinicians, and clinic staff as facilitators of video visits. For some, the disadvantages were sufficient to deter use of video visits, while for others, they were counterbalanced by the ease of access provided by video visits.

**Conclusions and Relevance:**

In this qualitative study, participants with NELP perceived multiple barriers to video visits, including greater communication difficulties, lower medical evaluation quality, and technical issues. These findings suggest that addressable technical challenges associated with language barriers hamper access to video visits and decrease motivation for use and that interventions are needed to increase telehealth equity.

## Key Points

**Question:**

What factors are perceived to lower use of video visits by patients with non–English language preference (NELP)?

**Findings:**

In this qualitative analysis of 27 interviews of patients with NELP, lower communication quality, lower medical evaluation quality, and difficulties with technical accessibility of video visits were reported to diminish motivation to use video visits. For some, these disadvantages were sufficient to deter use of video visits, while for others, they were counterbalanced by the ease of access provided by video visits.

**Meaning:**

These findings suggest that language barriers may decrease the use of video visits in multiple ways and that innovative interventions are needed to increase telehealth equity.

## Teaser

This qualitative study examines perceptions of facilitators and barriers to utilization of telehealth video visits among patients with non–English language preference.

## Introduction

Telemedicine, or synchronous video or audio-only visits, has made clinical encounters more convenient and accessible for many patients. Prior to the COVID-19 pandemic, video and telephone visits comprised a small minority of primary care visits,^1^ but by April 2020, approximately one-half of US physicians were treating patients virtually^2^ and nonurgent telemedicine video visits increased by more than 600%.^3^ While rates of telemedicine visits have decreased since the early COVID-19 pandemic, use remains substantially above pre–COVID-19 levels.^4,5^

Research on digital health tools such as electronic patient portals has shown lower uptake among racially and ethnically minoritized groups, older patients, patients with lower socioeconomic status, and patients with non–English language preference (NELP).^6,7,8^ Studies have also found that patients with NELP participate in video visits less often than their English-speaking counterparts.^9,10,11,12,13,14^ Structural barriers to telemedicine use by marginalized populations include lack of digital device and broadband access,^15,16^ limited telemedicine platform availability in languages other than English, and difficulties integrating interpreter services into telemedicine systems.^16,17^ However, one study^9^ found that video visit utilization rates remained lower for patients with NELP after adjusting for technology access, suggesting that additional factors may contribute.

One consideration may be the role of patient preference. The few published studies investigating preferences of patients with NELP for telemedicine have had inconsistent results.^18,19^ One study^20^ found that among those with lower use of video visits, including Spanish-speaking patients, practice-level and clinician-level factors accounted for more variance in video visit use than patient-level factors, suggesting intervention opportunities at the health system level.

To better understand the factors influencing utilization of video visits among patients with NELP, we conducted a qualitative study using the behavior change wheel and capability, opportunities, and motivation (COM-B) theory of health behavior^21,22,23,24,25^ as a conceptual model. This model identifies capability (physical and psychological capacities), opportunity (external social and physical factors), and motivation (internal automatic and reflective processes) as 3 key domains associated with health-related behaviors; the model also includes 9 intervention functions to produce behavior change.

## Methods

This qualitative study was approved by the University of California, San Francisco institutional review board and the reporting follows the Standards for Reporting Qualitative Research (SRQR) reporting guidelines.^26^ Researchers included 3 fluent Spanish-speakers (F.R.F., M.O.R., and A.F.) and 1 fluent Cantonese speaker (M.K.), 4 primary care clinicians (M.K., T.C.T., K.G., and A.F.), 1 postdoctoral scholar (B.I.), and 3 clinical administration leaders (L.B., T.C.T, and C.Y.).

### Study Design and Setting

We conducted a qualitative study using in-depth semistructured interviews with patients who preferred Spanish or Cantonese (the 2 most common non-English languages) in the ambulatory clinic network of a large, urban, academic health system serving a diverse population in the California San Francisco Bay Area. In this health system, patients with NELP represent 12.2% of the overall patient population (58 207 of 477 194 patients). Encounter data reveal that among patients with NELP, use of video visits relative to in-person visits has been approximately 10% lower than that of patients who preferred English since March 2020, with this difference persisting even as overall use of telemedicine visits has risen and fallen over time.

We selected 3 clinics with video visit utilization differences between patients who prefer English and patients with NELP, comparable to the difference for all ambulatory practices, to serve as the study recruitment sites: 1 adult internal medicine primary care clinic, 1 family medicine primary care clinic, and 1 adult gastroenterology specialty clinic. Among the 3 selected clinic sites, patients with NELP used video visits for a monthly average of 16% (125 of 781 video visits), compared with 32.2% (2135 of 6630 video visits) for patients who preferred English.

### Participants

Electronic health record data were used to identify patients with NELP who spoke Spanish or Cantonese and had at least 1 visit with the participating clinic but no video visits in the past 12 months. We did not ascertain from the electronic health record whether participants might have had a video visit before this 12-month period. For the primary care sites only, patients also needed to have a diagnosis of hypertension or diabetes because these conditions usually require regular follow-up while being clinically appropriate for video visits.^27,28,29^ Lists of eligible patients were sent to primary care clinicians to confirm eligibility. Spanish or Chinese language handouts about the study were mailed to eligible patients who were then called and offered participation in the study.

### Data Collection

The interview instrument (eMethods in Supplement 1) was designed by study team members (M.K., F.R.F., and A.F.) with expertise in language-concordant, patient-centered interviewing among limited-literacy populations. One-on-one semistructured interviews were conducted by language-concordant research staff (F.R.F., M.O.R., and M.K.) by telephone between November 2022 to September 2023 until thematic saturation was reached. Interviews were audio-recorded and deidentified, transcribed and translated by commercially available professional transcription and translation companies, and reviewed for accuracy by the language-concordant research staff who conducted the interviews. Verbal consent was obtained at the time of the interview.

### Data Analysis

Interviews were analyzed using rapid qualitative analysis, which is a recommended approach when information is needed to guide and improve implementation processes.^30^ Research team members (M.K., B.I., and F.R.F.) developed a structured template based on the interview guide to synthesize concise summaries of interview responses along with examples of illustrative quotations. Interview responses were aggregated onto a cross-participant analytic matrix, with topics comprising the columns and each participant listed in a row. To calibrate between team members, pairs independently summarized an initial subset of interviews using the template and then met to identify and resolve discrepancies.

The resulting matrix was analyzed to identify cross-cutting themes across participants.^31^ A group consensus process involving investigators from different disciplinary backgrounds was used to increase the rigor of data interpretation.^32^ Common themes were identified, matched to relevant domains of the COM-B model, and reviewed to describe areas of convergence and divergence across participants. Final themes were selected based on prevalence among participants and relevance to video visit utilization. Data analysis was conducted using from September 2023 to February 2024.

## Results

Of the first 31 patients who were successfully contacted by telephone, 27 (mean [SD] age, 66 [15] years; 18 women [67%]; 16 Spanish-speaking [59%]; 11 Cantonese-speaking [41%]) agreed to participate, leading to an 87% participation rate. Participant characteristics are shown in Table 1. Most participants reported having a phone, computer or tablet with internet, or cellular data access (20 participants [74%]). A wide range of digital literacy levels was reported. Some participants used their phones only for phone calls, others were able to answer video calls but not start them, and others used browsers, email, Youtube, or social media apps like WhatsApp, WeChat, and Facebook. Some participants had used Zoom or other video conferencing software, such as for a church activity or community-based support group. A few participants had used their clinic’s electronic health record patient portal and a few had used online translation apps to translate English text or portal messages. Many participants reported that their family members provided assistance with video visits, while a few reported getting support from a senior center, church, or community group.

**Table 1.** Participant Characteristics

Characteristic	Participants, No. (%) (N = 27)
Preferred language	
Spanish	16 (59)
Cantonese	11 (41)
Age, y	
Mean (SD)	66 (15)
Median (range)	67 (37-100)
Gender identity	
Woman	18 (67)
Man	9 (33)
Recruitment site	
Primary care	20 (74)
Specialty care	7 (26)
Has ever had a video visit	
Yes	14 (52)
No	13 (48)
Smart device access, yes	21 (78)
Internet or data access, yes	20 (74)
Stated preference for visit type	
In-person	15 (56)
Video	3 (11)
No clear preference	9 (33)

Participants described a variety of past experiences with video visits; approximately one-half had experienced a video visit in the past (14 participants [52%]). Some participants had been offered video visits but declined due to not knowing how to access them or not having a device, and others had attempted video visits but had difficulty connecting. Four major themes emerged (Table 2).

**Table 2.** Themes, Subthemes, Representative Quotations, and Relevant COM-B Model Constructs

Themes and subthemes	Quotations
Theme 1: video visits creating additional communication challenges and potentially exacerbating communication difficulties for patients who face language barriers (COM-B: motivation)	
Communication quality problems felt to be worsened or more difficult with video	“I would feel more comfortable talking to [the physician] in person. I could say whatever I like. If via Zoom, I have to think about it first, think well and be prepared … in person I feel more comfortable talking. It’s easier to talk more naturally … If I have questions, it’s easier to ask.” (Cantonese)
	“If you don’t have a good relationship with your primary care, it can be very cold and sometimes you need to make that connection ... the chemistry with that professional is very important, and sometimes virtuality could be an obstacle to that.” (Spanish)
	“Things are explained more clearly in person … It feels a little awkward when you see a doctor by video.” (Cantonese)
Continuity and language concordance with the clinician as facilitators for communication	“I’ve seen my family doctor for 20 years … once I hear the voice [on a telephone visit], I know it’s that doctor, it didn’t have to be awkward. And that doctor also speaks our language … We’ve seen each other [in person before], there’s a sense of familiarity, we can talk about anything.” (Cantonese)
	“I see that we need more doctors who speak Spanish ... I think it would be important to hire more bilingual staff so … [patients] feel accepted. When elderly people, for example, go to a visit they need that to build that trust with the doctor.” (Spanish)
Theme 2: video visits perceived as having some drawbacks for medical evaluations as well as some appealing benefits (COM-B: motivation)	
Video being seen as inferior for medical evaluations	“I think the average person likes seeing a doctor a face to face. The reason is sometimes it’s clearer … the doctor can check the patient with their own hands … he can check my heart, liver, spleen, lungs, and kidneys. Video visits are simply a one-on-one chat, the doctor can’t hear or know what’s going on inside my body at all.”(Cantonese)
	“I would like to see my primary doctor in person so that I could talk with her and she could examine me and touch me where it hurts … When we had a video call, I told her I had a welt in my nose and she said, ‘Well, I can’t see it very well.’ You can’t see well on a video call.” (Spanish)
	“If there are any major decisions or things like that, I wouldn’t want to do a video visit. If I know there’s nothing particularly serious, I can video chat with the doctor, or in the case of a follow-up, it’s okay to use video visits. But if now we are about to discuss why my heart is beating abnormally, or there are some new conditions that need surgery or something like that, I probably wouldn’t like using video visits very much because those things may be more important. And I’d hope to really have a time for it to be thought through clearly.” (Cantonese)
Positive qualities of and uses for video visits	“What I like is the convenience that you don’t need to ask for a day off from your job because you can take it anywhere you are. That is very convenient for me because you don’t need transportation, especially for people who don’t have a car or who have difficulty moving around. It saves time and money.” (Spanish)
	“Video visits have an advantage. There is no need to go directly to the hospital because it takes half an hour to park the car to go to the hospital. There is still a possibility that one can’t go to the hospital because of work or other circumstances even if you’ve made an appointment … But for a video visit, even if you go to work at most, in the appointed period you just walk away to finish that video visit. It can be done without affecting work or with little impact, and this is good.” (Cantonese)
	“If I need to be examined due to some rash or a wound I might have, it would be preferable to do it in person. But if you only need to talk to him, if I saw him two weeks ago or a month ago, and it’s not something urgent, and I only need to tell him I need a higher dose of my medication, or he just needs to send me to the lab to do some tests, I don’t need to see him in person … we can discuss it virtually.” (Spanish)
Theme 3: limited digital literacy, device and data access, and non–user-friendly video visit processes as important barriers to video visit use. (COM-B: capability and opportunity)	
Limited digital literacy, especially for older adults, as a substantial barrier	“Last time my son set it up … My son said just press on that place and that’s all you have to do … After pressing it, I didn’t know how to work it, didn’t know what to say. I just saw the images moving back and forth, there was a man moving, and a woman moving, and had to go through the interpreter, right? After [it] messed around for a long time, then the interpreter finally said you can talk, we can hear. So it was very troublesome. We don’t know how to use it, most importantly. Probably the younger [patients], it’s no problem. People of a certain age, we don’t know this new technology, right?” (Cantonese)
	“That’s the problem with the video visits. On the phone it’s different, but one day they told me, ‘You have a video visit and the doctor doesn’t want to have a phone call because he needs to verify your identity so that the insurance can pay. They need to see the person.’ But insurance companies should take that into account, that there are elderly people from different countries and cultures because this is a multicultural country, so they should take into account that elderly people don’t have very good knowledge of technology compared to younger people.” (Spanish)
Process not user friendly, too many steps, and troubleshooting too difficult to access if attempt is not working or there is need to reconnect	“The problem is that many of us who are elderly are not very familiar with the technology … Zoom sometimes fails and you have to call again and that’s when I have problems with the reconnection … When I have a video visit I feel anxious … if you don’t know how to use it well, I’m afraid the connection will fail and then I won’t be able to connect again and I will lose my appointment. Doctors and practitioners are very busy people and they have a specific time assigned to you so, they won’t be dealing with you if your connection cuts off.” (Spanish)
	“I need some family members to help me, I can’t do it until the video is ready … I don’t know how to operate it, I mean I don’t know how to use a computer ... I don’t understand English and I don’t know how to use the code [to get into the video visit].” (Cantonese)
Cell phone data plans and service issues and phone or internet costs as barriers	“Sometimes, many of us use plans that are not very expensive … my cell phone [plan] doesn’t have a good service. I use the cheapest plan they have, and many of us, the seniors, are in the same situation. [There isn’t] good reception in certain places. The calls cut off.” (Spanish)
Theme 4: in-person teaching, simpler technologic processes, opportunities to repetitively practice video use, troubleshooting support, and language-concordant instructions, clinicians, and clinic staff as facilitators of video visits. (COM-B: opportunity)	
Assistance from family members, the clinic, or other supports, especially in-person or direct assistance	“While I was talking with the doctor, Zoom cut off, but as my daughter was here she was able to connect again … Before the pandemic hit I went to the Senior Center where I received computer classes. It would be great to be near the computer lab when I have the Zoom appointment because there was a person present there who could assist us.” (Spanish)
	“I think those materials sent by [the clinic] are pretty good. It teaches you how to do it or when you made an appointment for a video visit … It tells details like what you should press and then what will appear and who you should call when you have any more questions … Especially the first time, someone will remind you how to do it before that, asking if you know how to do it, and they keep asking.” (Cantonese)
Simpler processes, in-person teaching, opportunities to practice repetitively, increased troubleshooting support, and language concordance as helpful resources	“I think they should organize workshops for people. Nurses or people who … visit them at home and explain it to them and show them how to use the applications. ‘Look, you only have to press this’ … if you throw someone into the water and he doesn’t know how to swim, he will drown, but if you teach him how to swim before throwing him into the water, he won’t get drowned. I think that training is very important. I’m not a fan of technology but I think we have to recognize that technology can make our lives easier if we know how to use it, if we know how to apply it.” (Spanish)
	“The best way is [for the clinic] to get a sample [visit link] ready, [send it] by just one click ... the simpler you make it, the better … If you want to make it popular and simple, don’t think about things from a young person’s perspective. It’s different for the elderly.” (Cantonese)

Abbreviation: COM-B: capability, opportunities, and motivation model.

### Theme 1: Video Visits Creating Additional Communication Challenges and Potentially Exacerbating Communication Difficulties for Patients Who Face Language Barriers (Motivation)

Many participants perceived reduced communication quality during video visits as a motivational barrier. They reported that it felt harder to ask questions or explain their symptoms over video and that communication was less direct, comfortable, or natural:

“[In-person,] I just say what I have to say, it’s more natural … it’s easier to communicate. It’s hard enough to use an interpreter, adding video makes communication even more difficult.” (Cantonese)

Some participants described not being able to see gestures, body language, or expressions as a contributor to their concerns about video visits. A few reported that it would be harder to speak up during a video visit, that they would have to think harder about what to say, or that there would be less mental space to think about what is communicated:

“Sometimes … you want to say something and you can’t … you explain things, but how are they supposed to understand you?” (Spanish)

A few participants described perceiving they would get more attention or respect during in-person visits compared with video. Concerns about communication were more often reported among Cantonese-speaking than Spanish-speaking participants. In contrast, some patients reported positive experiences with video visits in the past and felt that the communication quality was similar to in-person visits. Some participants mentioned having a continuous relationship and language concordance with their clinician as facilitators for communication.

### Theme 2: Video Visits Perceived as Having Some Drawbacks for Medical Evaluations as Well as Some Appealing Benefits (Motivation)

Among participants who reported a preference for in-person visits (15 participants [56%]), many believed that video visits may not provide as sufficiently comprehensive or appropriate of evaluations as in-person visits. Several mentioned lack of physical examinations or not being able to get vital signs or tests done as reasons for believing the visit would be less comprehensive:

“They can’t examine you … I don’t think it’s right to just talk to the doctor and not be examined to be able to see what the problem is.” (Spanish)

Others stated they would not be confident in a video visit for a new, urgent, or complex problem because something could be missed. One participant described video visits as not feeling like a real visit:

“It can be real only when you see a doctor in person.” (Cantonese)

At the same time, most patients identified positive aspects of video visits. Convenience, saving time, avoiding transportation or parking, not needing to take time off work, and not needing to travel with poor mobility, during poor weather, or when feeling ill were commonly perceived advantages. Several participants reported that video visits would be appropriate for follow-up visits, visits mostly involving discussion, or simple issues not requiring an examination. A few participants also mentioned that it seemed easier to get an appointment sooner over video.

### Theme 3: Limited Digital Literacy, Device and Data Access, and Non–User-Friendly Video Visit Processes as Important Barriers to Video Visit Use (Capability and Opportunity)

Limited digital literacy, especially for older adults, was frequently described as a barrier to video visits. A few participants described anxiety around video visits due to not knowing what to expect or how to troubleshoot:

“I didn’t know what was going to happen. Why didn’t the doctor show up? Is it time yet? Could it be that the doctor forgot to see me? Or do I need to make a phone call? I was anxious.” (Cantonese)

Several participants discussed digital access as being particularly difficult, confusing, or troublesome for older people without basic technology skills. Several noted memory issues or difficulty learning new skills, and said they would need ongoing reminders or repetition to learn how to use video visits:

“I don’t know how to use Zoom or that type of thing. I don’t feel I have the ability to do it, that’s why I don’t do it. …I need instructions although I don’t have an open mind to remember … it goes in one ear and out the other.” (Spanish)

Many participants also described the video visit process as not user friendly, requiring too many steps, and being difficult to access without troubleshooting assistance. A few described being able to do 1-step processes with their devices, but that processes requiring an extra step, such as using a password, were too complicated. A few reported that family members or their clinic team did not have enough time or availability to help them. Some described having inadequate or outdated devices, and a few participants discussed the costs of reliable data or internet plans as barriers.

### Theme 4: In-Person Teaching, Simpler Technologic Processes, Opportunities to Repetitively Practice Video Use, Troubleshooting Support, and Language-Concordant Instructions, Clinicians, and Clinic Staff as Facilitators of Video Visits (Opportunity)

Many participants reported video visit assistance from family members, the clinic team, or community-based sources as helpful, especially when the assistance was in person. Some reported appreciating their clinic teaching them in person on how to use video visits, giving them reminder calls or video-visit instructions with visuals, and installing the needed apps for them beforehand.

Resources that participants felt would be helpful included simpler processes, with the ideal being a simple, 1-click process:

“If I had a tablet that was already configured to use [the video visit], I would simply click and make the call.” (Spanish)

Repeated teachings, demonstrations, and opportunities to practice were a common suggestion, especially if in-person and in the person’s preferred language:

“One attempt only is not enough, it really takes several times to get the skill.” (Cantonese)

One participant suggested a short language-concordant video to demonstrate how to log on before a video visit. Others reported they would like troubleshooting assistance such as knowing where to call when having issues or having an assistant by their side to directly support them. Making sure instructions are in patients’ preferred languages, having high-quality interpreters readily available, and more bilingual staff and clinicians were also suggested.

## Discussion

In this qualitative study, we found that patients with NELP perceived several barriers to video visits including communication quality, medical evaluation quality, and technical accessibility. The additional communication barriers on top of pre-existing communication challenges from language discordancy, as well as concerns about inferior medical evaluations using video, diminished the motivation of patients with NELP to use video visits. While patients who prefer English also face barriers with technical accessibility,^15^ the additional concerns about quality of communication and medical evaluation with video visits in the context of language discordance with clinical teams represent unique barriers for patients with NELP. For some patients with NELP, these additional perceived concerns are sufficient to inhibit motivation and prevent attempts to overcome digital literacy and technical accessibility barriers to use video visits. For others, these concerns may be sufficiently counterbalanced by the attractive aspects of video visits. We depict these motivational factors and how they can interact with each other in the Figure.

**Figure.**
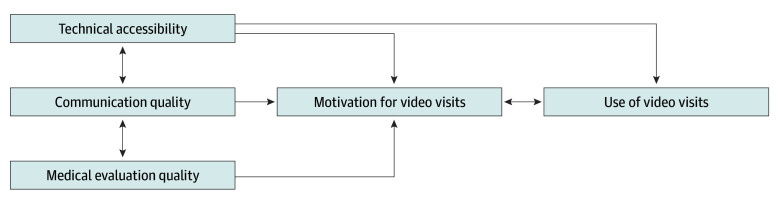
Perceived Factors Affecting Motivation to Use Video Visits Among Patients With Non-English Language Preference Arrows represent the interaction between perceived factors and motivation to use video visits among patients with non-English language preference.

On the surface, lower patient motivation for video visits may seem to represent a matter of preference among patients with NELP that is separate from structural barriers limiting video visit usage. However, our findings highlight a more nuanced interplay between technical accessibility, motivation, and video visit usage. Structural barriers that reduce technical accessibility for patients with NELP influence motivation in addition to directly limiting video visit usage. Thus, lower motivation for video visits, while associated with lower video use, is not an isolated, intrinsic preference. Rather, motivation is shaped by systemic language and digital access barriers that influence patient expectations and engagement with health care; this is consistent with other analyses describing the difficulty of separating patient preferences in health care from the influence of historically inequitable experiences due to structural factors and how preference may represent resignation to the perceived status quo of inaccessible or lower-quality interventions.^33,34^

Studies have found video visits to offer improved communication, ability to visually demonstrate health conditions, diagnostic accuracy, blood pressure and depression screening, and higher satisfaction compared with telephone visits.^20,35,36,37,38,39,40^ In addition, prior research has found interest in video over telephone formats among safety-net patient populations despite barriers to video access.^18,19^ For patients potentially motivated to have video visits, maximizing accessibility of video visits remains important so that patients with NELP who already have barriers to health care access^41^ can meaningfully and equitably benefit from advances in health care technology.^42,43^

While device, internet, and data availability^15,17^ and linguistic accessibility of electronic health record and visit interfaces^16,17^ are important large-scale structural issues to address, our findings suggest ways to shrink accessibility gaps that are within the control of a health system. We recommend health system–level interventions in 4 areas, and map these to COM-B intervention functions in Table 3:

**Table 3.** Recommendations and Relevant COM-B Model Constructs

Recommended interventions	COM-B intervention functions
Work to create as much of a passive, 1-click process for video visits as possible Advocate and codesign with platforms and vendors Streamline and reduce unnecessary steps Assist patients with preconfiguring devices	Environmental restructuring
Provide interactive demonstration and practice opportunities In-person teaching and workshops preferred for older patients or patients with limited digital literacy Consideration of videos over printed materials and visuals over text Repetition of content needed	Education, training, and modeling
Increase onboarding and troubleshooting supports with a navigator or as-needed assistant Create workflows for equitable screening and ask patients when they come for visits about readiness and interest Explore opportunities for in-person elbow-to-elbow support for early attempts (eg, from family, clinic staff, community-based organizations, or others) Create and publicize a support number to call if having difficulty	Education, training, modeling, and enablement
Maximize language concordance at each step of communication Review and increase concordance of texts and notifications, patient portal interface, and video visit instructions Improve quality and availability of interpreters Increase availability of language concordant staff and clinicians Advocate for linguistic accessibility of electronic health records and portals	Environmental restructuring

Abbreviation: COM-B, capability, opportunities, and motivation model.

1. Create as simple of a 1-click process for video visits as possible. Each added step to access a video visit introduces potential difficulty, especially for those with limited digital literacy and those without language-concordant interfaces; this may involve codesign with interface vendors and simplifying clinic processes to be more user-friendly. Streamlining and removing unnecessary steps or clicks, having ways to push a 1-click link to patients (such as via text message), and assisting patients with preconfiguring their devices are potential strategies.
2. Provide repetitive, visual, interactive demonstrations and practice opportunities to train patients with limited digital literacy to use video visits. In-person teaching was felt to be most effective, and video- and visual-based materials were considered to be more useful than text-only instructions.
3. Provide an as-needed, language-concordant assistant or navigator^44,45^ to help assess patients for video-visit support needs, train those needing additional help, and provide easily accessible language-concordant technical assistance if patients encounter difficulties during video visits.
4. Maximize language access at every level and stage of communication with patients; this includes having language-concordant notifications, portals, and video visit interfaces; improving the accessibility and quality of interpreters during video visits; and increasing overall availability of language-concordant staff and clinicians.

### Limitations

This study has limitations. While this study was conducted in 1 delivery system, generalizability is suggested by themes that were also found in other settings.^16,17,19^ This study adds perspectives on barriers for patients with NELP in an academic health system with diverse payors to prior qualitative research done primarily in safety-net settings. Our study only included Spanish- and Cantonese-speaking patients; other language and cultural backgrounds may experience fewer, greater, or different challenges. Comments about communication quality were more heavily represented among Cantonese-speaking participants in this study, suggesting that additional factors may potentiate communication-quality concerns for specific language groups. We studied patients from a gastroenterology specialty clinic and those with hypertension or diabetes from primary care clinics; other considerations may be found among different patient populations.

## Conclusions

Technological innovations provide important benefits in health care. However, patient populations with greater barriers to access and meaningful use of these innovations are at risk of being left behind in an inequitable digital divide. Our qualitative study did not find an inherent universal objection to video visits among patients with NELP, but that like all patients, patients with NELP have a range of perspectives ranging from being never, to sometimes, to highly open to video visits. All would like the opportunity to make thoughtful decisions about their use of video visits.

Our study adds to the literature by highlighting patient perspectives on the relationship between technical accessibility and motivation to use video visits. Simple video visit processes and strategies for supporting patients with NELP are needed. Further research should evaluate the efficacy of health system efforts to provide language accessibility, targeted video visit supports, and patient-centered telemedicine processes. Such efforts will allow health systems to better address the needs of diverse patient populations while accommodating a spectrum of motivations and capabilities.

## Notes

Supplement 1. **eMethods.** Interview Guide

Supplement 2.
Data Sharing Statement
