# Supplementary material for: Challenges to Video Visits for Patients With Non–English Language Preference: A Qualitative Study
Source: JAMA Netw Open. 2025 Feb 12;8(2):e2457477. doi: 10.1001/jamanetworkopen.2024.57477 (PMC11822542; doi:10.1001/jamanetworkopen.2024.57477)
Supplement: Supplement 1. — eMethods. Interview Guide [file jamanetwopen-e2457477-s001.pdf]

Supplemental Online Content

Kong M, Rios-Fetchko F, Olmos-Rodriguez M, et al. Challenges to video visits for patients with non-English language preference: a qualitative study. *JAMA Netw. Open*. 2025;8(2):e2457477. doi: 10.1001/jamanetworkopen.2024.57477.

eMethods. Interview Guide

This supplemental material has been provided by the authors to give readers additional information about their work.

© 2025 Kong M et al. *JAMA Network Open*.

eMethods. Interview Guide

1) Have you heard that your clinic offers video visits to see the doctor?
    a. What have you heard about it?
    b. How do you usually see your doctor? Is there usually an interpreter offered?

2) Have you ever had a video visit before with any clinic or doctor before?

    a. If Yes: What was the experience like overall?
        i. What did you like about it?
        ii. What did you dislike about it?
        iii. How did the visit work with the interpreter?
        iv. What was it like to sign into a video visit on the computer or phone?
        v. Did anyone help you get ready for the video visit, like by helping set up your device?

    b. If No: Have you been offered to do a video visit before? If so, what made you decide to do it (or not)?
        i. Did the clinic staff offer help or resources with setting up and learning to use a video visit?
            1. If yes, tell us about that process and how you felt about it.
        ii. If you said no to doing the video visits, tell us about wh
        iii. y you decided to decline.
        iv. If you were to imagine a video visit with your doctor, what do you think it would be like? (Probe: quality of care, communication, getting health needs met, interpreter usage)

3) Some people choose to do video visits, and some don't. What do you think would make someone **want** to do a video visit (instead of in-person visit)?
    a. What could be easier about having a visit over video (compared to in-person)?

4) What do you think makes people **not want** to do a video visit?
    a. What would be harder about having a visit over video (compared to in-person)?

5) What would an ideal video visit look like?

6) How often are you using apps/video on your phone/tablet in general?
    a. What do you feel it would be like for you to try signing in to a video visit on a computer or phone? Why?

7) What kind of help would you want or need to have a video visit?
    a. Who would you expect to provide that help, and what kinds of things would you want them to do to help?

8) What type of visit would you prefer in the future? Why?
    a. In what situations would you prefer an in-person visit? Phone? Video?
    b. Would you be interested in trying a video visit to see your doctor? Why or why not?

9) What other comments, thoughts, or experiences related to video visits would you like to share?

10) Lastly, if you're able to let us know some demographic data, that would help us know how well our interviews represent the clinics' patients overall.
    a. What is your age?
    b. What is your gender identity?
    c. How would you describe your level of comfort or ability with English (very well, well, not well, not at all)?
    d. Do you have a long-term health condition, like high blood pressure or diabetes?

© 2025 Kong M et al. *JAMA Network Open*.
