# Supplementary material for: Challenges to Video Visits for Patients With Non–English Language Preference: A Qualitative Study
Source: JAMA Netw Open. 2025 Feb 12;8(2):e2457477. doi: 10.1001/jamanetworkopen.2024.57477 (PMC11822542; doi:10.1001/jamanetworkopen.2024.57477)
Supplement: Supplement 2. — Data Sharing Statement [file jamanetwopen-e2457477-s002.pdf]

Data Sharing Statement

Kong. Challenges to Video Visits for Patients With Non–English Language Preference. *JAMA Netw Open*. Published February 12, 2025. doi:10.1001/jamanetworkopen.2024.57477

Data
Data available: No
